# Anorectal lymphogranuloma venereum among men who have sex with men: a 3-year nationwide survey, France, 2020 to 2022

**DOI:** 10.2807/1560-7917.ES.2024.29.19.2300520

**Published:** 2024-05-09

**Authors:** Olivia Peuchant, Cécile Laurier-Nadalié, Laura Albucher, Carla Balcon, Amandine Dolzy, Nadège Hénin, Arabella Touati, Cécile Bébéar, on behalf of the Anachla study group

**Affiliations:** 1 Bordeaux University Hospital Center, Department of Bacteriology, National Reference Centre for bacterial Sexually Transmitted Infections, Bordeaux, France; 2 Univ. Bordeaux, UMR 5334 CNRS Microbiologie Fondamentale et Pathogénicité (MFP), ARMYNE, Bordeaux, France; 3 Members of the Anachla study group are listed under Acknowledgements

**Keywords:** *C. trachomatis*, LGV, lymphogranuloma venereum, anorectal symptoms, PrEP, MSM, *omp*A genotype

## Abstract

**Background:**

In France, lymphogranuloma venereum (LGV) testing switched from universal to selective testing in 2016.

**Aim:**

To investigate changes in LGV-affected populations, we performed a nationwide survey based on temporarily reinstated universal LGV testing from 2020 to 2022.

**Methods:**

Each year, during three consecutive months, laboratories voluntarily sent anorectal *Chlamydia trachomatis*-positive samples from men and women to the National Reference Centre for bacterial sexually transmitted infections. We collected patients’ demographic, clinical and biological data. Genovars L of *C. trachomatis* were detected using real-time PCR. In LGV-positive samples, the *omp*A gene was sequenced.

**Results:**

In 2020, LGV positivity was 12.7% (146/1,147), 15.2% (138/907) in 2021 and 13.3% (151/1,137) in 2022 (p > 0.05). It occurred predominantly in men who have sex with men (MSM), with rare cases among transgender women. The proportion of HIV-negative individuals was higher than that of those living with HIV. Asymptomatic rectal LGV increased from 36.1% (44/122) in 2020 to 52.4% (66/126) in 2022 (p = 0.03). Among users of pre-exposure prophylaxis (PrEP), LGV positivity was 13.8% (49/354) in 2020, 15.6% (38/244) in 2021 and 10.9% (36/331) in 2022, and up to 50% reported no anorectal symptoms. Diversity of the LGV *ompA* genotypes in the Paris region increased during the survey period. An unexpectedly high number of *ompA* genotype L1 variant was reported in 2022.

**Conclusion:**

In rectal samples from MSM in France, LGV positivity was stable, but the proportion of asymptomatic cases increased in 2022. This underscores the need of universal LGV testing and the importance of continuous surveillance.

**Key public health message**

**What did you want to address in this study and why?**

In France, universal lymphogranuloma venereum (LGV) testing from 2010 to 2016 showed that LGV was associated with anorectal symptoms and living with HIV infection. Thus, since 2016, screening for LGV has been limited to people living with HIV and/or with anorectal symptoms, leading to biased data about LGV epidemiology. We investigated changes in LGV epidemiology through temporary universal LGV testing in 3 months of 2020, 2021 and 2022.

**What have we learnt from this study?**

The LGV positivity among *C. trachomatis*-positive anorectal specimens remained stable over the 3 years. Individuals diagnosed with LGV were more often asymptomatic than in the past, and the proportion of HIV-negative cases was higher compared with those living with HIV. Among PrEP users, LGV positivity did not vary over the studied period, and up to half did not report anorectal symptoms.

**What are the implications of your findings for public health?**

In France, the characteristics of LGV-affected populations have changed since the beginning of the epidemic. All *C. trachomatis*-positive anorectal specimens should be tested for LGV, regardless of HIV status and anorectal symptoms.

## Introduction

Lymphogranuloma venereum (LGV) is an invasive and ulcerative sexually transmitted infection (STI) caused by genovars L of *Chlamydia trachomatis*. It has been endemic in Europe since 2003. The number of LGV cases reported has risen year on year, from 739 in 2011 to 3,112 in 2019 in the 23 European Union/European Economic Area (EU/EEA) countries where enhanced LGV surveillance was implemented [1]. Notably, the number of cases reported in Europe in 2019 had increased by 30% compared with 2018. The majority of cases were reported in Belgium, France, the Netherlands, Spain and the United Kingdom (UK). Affected were mainly high-risk groups of men who have sex with men (MSM), those living with HIV and individuals who are part of dense sexual networks with high rates of partner change, with proctitis as the most common manifestations [2]. In some countries, changes in testing recommendations for LGV were followed by increased diagnosis of LGV cases among HIV-negative MSM and/or asymptomatic MSM [3-5]. For the screening policies in three selected countries, please refer to the appended Supplementary Table S1.

In line with changes in testing practices in some EU/EEA countries, the updated European guidelines on the management of LGV recommended in 2019 that all MSM with anorectal specimens positive for *C. trachomatis* should be tested for LGV irrespective of symptoms [2]. The identification of L genovars in *C. trachomatis*-positive anorectal samples is recommended because therapy of extended duration is required. In France, a national LGV surveillance network was established at the French National Reference Centre (NRC) for bacterial sexually transmitted infections (STIs) in 2010. In this sentinel network, laboratories in the metropolitan area and overseas territories perform routine testing for *C. trachomatis* detection in anorectal specimens and send *C. trachomatis*-positive specimens to the NRC for molecular LGV diagnosis [6]. Data collected in France from 2010 to 2015 showed that LGV was associated significantly with rectal symptoms and people living with HIV [6]. Based on these results, the NRC has been performing selective LGV testing only for patients with anorectal symptoms and/or HIV since 2016. Changes in the indications for LGV testing have complicated the interpretation of LGV positivity over time.

The LGV *omp*A genotypes are used to investigate the dynamics and evolution of LGV. In the last decade, some countries reported a shift to *omp*A genotype L2/434/Bu predominance, as well as the emergence of other *omp*A genotype L2b variants [7-9]. Recently, a recombinant strain, the L2b/D-Da hybrid variant, was identified; whole-genome sequencing showed that some of these hybrid variant strains carried the Ser83Ile amino acid change in DNA gyrase subunit A (GyrA), which is associated with fluoroquinolone resistance [10,11].

To investigate the epidemiology and characteristics of LGV-affected populations since the discontinuation of universal screening in 2016, we conducted a 3-month prospective nationwide survey in 2020, 2021 and 2022 in which *C. trachomatis*-positive anorectal specimens were tested for LGV, irrespective of HIV status and anorectal symptoms.

## Methods

### Specimen collection

Each year, during three consecutive months, laboratories in mainland France and overseas territories voluntarily sent *C. trachomatis*-positive anorectal samples to the NRC for specific LGV real-time PCR analysis. As soon as the results were available, the NRC invited the prescribing clinicians to complete a standardised questionnaire to provide patients’ demographic (gender – men, women, transgender, unknown –, year of birth and department of residence), biological (HIV status and concurrent STIs), clinical (anorectal symptoms) and sexual orientation data. These clinicians obtained patients’ written informed consent and sent the completed questionnaires to the NRC [6].

For the present study, the specimens were collected from 1 March to 31 May in 2021 and 2022. In 2020, because of the national lockdown due to the COVID-19 pandemic during these months, specimens were instead collected from 1 September to 30 November. We analysed epidemiological data obtained between 2020 and 2022 from the 59 laboratories that participated in the 3 years of the survey. The commercial assays used for *C. trachomatis* detection in these laboratories were the following: Abbott RealTime CT/NG, Alinity m STI AMP Kit (Abbott), Allplex CT/NG/MG/TV Assay (Seegene), Allplex STI essential assay (Seegene), Aptima Combo 2 (Hologic), BD MAX CT/GC (Becton-Dickinson), cobas CT/NG (Roche), STI PLUS ELITe MGB kit (ELITech), Xpert CT/NG (Cepheid), *N. gonorrhoeae*/*C. trachomatis*/*M. genitalium*/*T. vaginalis* Real-TM kit (Sacace Biotechnologies). Only one sample per patient per year was considered for analysis.

As universal LGV testing was performed in France until 31 December 2015, we compared risk factors for LGV in 2015, 2020, 2021 and 2022 using data from the same 28 laboratories that voluntarily participated in the survey in those years. In 2015, specimens and data had been collected as described above [6].

### Laboratory testing and case definition

DNA was extracted from specimens using the MagNA Pure 96 instrument (Roche Diagnostics). All anorectal specimens contained an extraction and inhibition real-time PCR internal control (DICD-CY-L100; Diagenode Diagnostics).

For LGV diagnosis, we used a genovar L-specific real-time PCR assay that targeted a unique gap of 36 bp in the *pmp*H gene [12]. The positivity of LGV was calculated as the number of subjects diagnosed with LGV divided by the total number of subjects tested for LGV.

For all L genovar specimens, a 1,100 bp fragment of *omp*A gene was amplified by nested PCR and sequenced [13]. Sequences were compared by alignment with chlamydial LGV *omp*A gene sequences available in GenBank. We used the term *omp*A genotype to refer to strains identified by *omp*A sequencing [14]. A phylogenetic tree based on *omp*A nucleotide sequence was constructed using the maximum likelihood clustering method based on the Tamura 3-parameter model (T92 Q + I model) [15]. The MEGA software (version 7.0; University Park, US) was used for tree elaboration and the iTOL software (version 6.0; Heidelberg, Germany) was used for tree visualisation and annotation [16,17].

For specimens with LGV *omp*A genotypes, a 362 bp fragment of the *gyr*A gene encompassing the quinolone resistance determining region (QRDR) was amplified and sequenced using the primers CTA3 and CTA4 [11].

### Statistical analysis

Fischer’s exact test and the chi-squared test were used for qualitative variables, and analysis of variance and Student’s *t* test were used for quantitative variables. We considered p values < 0.05 to indicate statistical significance. We performed uni- and multivariate logistic regression analyses of risk factors associated with LGV using the RStudio software (version 4.3.0; 2023–04–21 ucrt). Variables that were previously identified as LGV risk factors using the French sentinel surveillance for anorectal *C. trachomatis* infections data between 2010 and 2015 were used for the multivariate model: residence (Paris region, other regions than Paris), anorectal symptoms (no, yes) and HIV status (negative, positive) [6].

## Results

In 2020, 2021 and 2022, respectively 88, 90 and 112 laboratories in the metropolitan area and overseas territories of France participated in the survey. Among them, 59 laboratories participated in all 3 years and provided 90% of the specimens in 2020 and 2021 and 78.6% in 2022. These laboratories were distributed throughout France.

We analysed a total of 3,191 *C. trachomatis*-positive anorectal samples (1,147 in 2020, 907 in 2021 and 1,137 in 2022) over the 3 years. Approximately 30% of the specimens were collected from the Paris region; we provided details on the geographical distribution of the samples in Supplementary Table S2. More than 90% of the specimens were obtained from men, and only a small number were from cisgender women: 3.9% (45/1,147) in 2020, 5.3% (48/907) in 2021 and 5.2% (58/1,137) in 2022. The LGV positivity was 12.7% (146/1,147) in 2020, 15.2% (138/907) in 2021 and 13.3% (151/1,137) in 2022 (p > 0.05), with no difference according to the month of the study; data by month are appended in Supplementary Figure S1. The mean age of patients providing the samples was significantly younger in 2021 than in 2020 and 2022 (p = 0.02) (Table 1). No case of LGV occurred in cisgender women. All but seven LGV men with known sexual behaviour described themselves as MSM. Most LGV diagnoses (65.5%, 285/435) were made in patients residing in regions other than Paris, and this distinction tended to decrease slightly over the study period, from 68.8% (95/138) to 58.9% (89/151) (p > 0.05) (Table 1). The LGV positivity varied by region but there was no difference over the study period within the same region; for detailed data on the regional distribution, we refer to Supplementary Table S2. Of the LGV cases with known HIV status (91.9%, 397/432), the proportion of HIV-negative individuals was higher than that of those living with HIV, but this difference decreased slightly over the study period, from 59.1% (81/137) in 2020 to 51.9% (69/133) in 2022. Asymptomatic rectal LGV increased from 36.1% (44/122) in 2020 to 52.4% (66/126) in 2022 (p = 0.03). This proportion fluctuated among HIV-negative people (p = 0.24) but increased from 35.6% (16/45) in 2020 to 60.9% (39/64) in 2022 among those living with HIV (p = 0.02); data by HIV status are appended in Supplementary Table S3.

**Table 1:** Characteristics of lymphogranuloma venereum cases, France, 2020–2022 (n =435)

LGV-positive cases	2020 (n = 146)		2021 (n = 138)		2022 (n = 151)		p value
	n	%	n	%	n	%	
Age (years)	n = 145		n = 136		n = 151		
Median (IQR)	39 (30.2–47.2)		33 (28–41.25)		41 (31.5–50)		**0.02**
Mean	39		36		41		
≤ 34	54	37.2	74	54.4	50	33.1	**0.0006**
35–39	22	15.2	21	15.4	20	13.2	0.84
40–44	18	12.4	14	10.3	23	15.2	0.45
≥ 45	51	35.2	27	19.9	58	38.4	**0.002**
Gender	n = 146		n = 137		n = 151		
Men	144	98.6	135	98.5	149	98.7	1
Transgender (male to female)	2	1.4	2	1.5	2	1.3	
Sexual orientation for men	n = 104		n = 73		n = 81		
MSM	103	99	69	94.5	79	97.5	0.21
Heterosexual	1	1	4	5.5	2	2.5	
Residence	n = 146		n = 138		n = 151		
Paris region	47	32.2	42	30.4	61	40.4	0.21
Other metropolitan French regions	99	67.8	95	68.8	89	58.9	
French overseas territories^a^	0	0	1	0.7	1	0.7	
HIV status	n = 137		n = 127		n = 133		
Living with HIV	56	40.9	53	41.7	64	48.1	0.44
Negative	81	59.1	74	58.3	69	51.9	
Anorectal symptoms	n = 122		n = 102		n = 126		
Yes	78	63.9	60	58.8	60	47.6	**0.03**
No	44	36.1	42	41.2	66	52.4	

IQR: interquartile range; LGV: lymphogranuloma venereum; MSM, men who have sex with men.

^a^ Martinique and French Guiana.

The total number of responses is indicated for each variable. p values in bold indicate significance.

**Figure 1:**
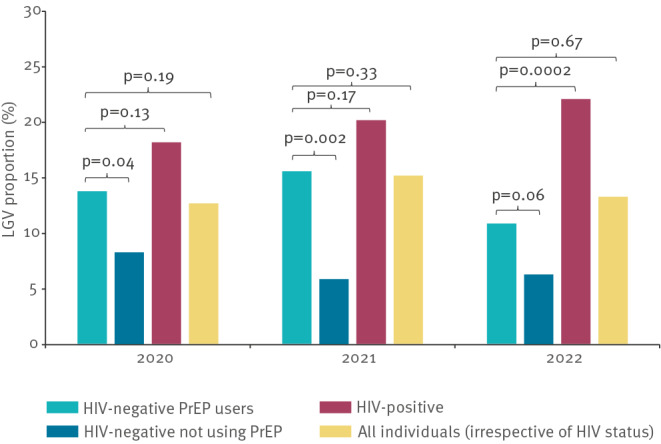
Lymphogranuloma venereum proportion, overall and by HIV status, PrEP use, France, 2020–2022 (n = 3,191)

LGV: lymphogranuloma venereum; PrEP: pre-exposure prophylaxis.

Most HIV-negative individuals with LGV were using HIV pre-exposure prophylaxis (PrEP). The LGV positivity among PrEP users was 13.8% (49/354) in 2020, 15.6% (38/244) in 2021 and fell to 10.9% (36/331) in 2022 (p = 0.24) (Figure 1). It was significantly higher than that in HIV-negative individuals not using PrEP, except in 2022. Conversely, the LGV positivity was similar among PrEP users and individuals living with HIV, except in 2022 (p = 0.002). Finally, the proportion of LGV cases was similar among PrEP users and the entire population (p > 0.05). Up to 50% of LGV-positive PrEP users reported no anorectal symptoms; for the numbers by symptoms see Supplementary Table S4. 

For HIV-negative individuals, data on PrEP use were lacking in 12 of 81 (14.8%) cases in 2020, 25 of 74 (33.8%) in 2021 and 19 of 69 (27.5%) in 2022.

### Comparison of data from 2015, 2020, 2021 and 2022

In 2015, 184 LGV cases were diagnosed. The median age of the patients was 39 years (interquartile range: 32–46). All were men, mostly MSM (118/119). Overall, 53.2% (98/184) of LGV cases in 2015 were from other regions than the Paris region, 86.6% (103/119) reported anorectal symptoms and 63.2% (91/144) were living with HIV. Univariate analyses showed that LGV was associated with residence in the Paris region in 2015 and 2022, but not in 2020 or 2021 (Table 2). The odds ratio (OR) for anorectal symptoms decreased in 2020 and 2022 compared to 2015 (albeit with overlapping confidence intervals), but not in 2021. The OR for HIV positivity decreased from 2015 to 2020–2022. The multivariate analysis confirmed these findings.

**Table 2:** Univariate and multivariate logistic regression analysis for lymphogranuloma venereum risk factors, France, 2015, 2020, 2021 and 2022 (n = 496)

Characteristics	2015		2020		2021		2022	
	Crude OR (95% CI)	Adjusted^a^ OR (95% CI)	Crude OR (95% CI)	Adjusted OR (95% CI)	Crude OR (95% CI)	Adjusted OR (95% CI)	Crude OR (95% CI)	Adjusted OR (95% CI)
Residence								
Other regions than Paris	1	1	1	1	1	1	1	1
Paris region	2.46 (1.61–3.76)	2.08 (1.19–3.76)	1.06 (0.70–1.61)	0.9 (0.57–1.44)	1.07 (0.65–1.77)	0.95 (0.54–1.66)	2.12 (1.43–3.16)	2.01 (1.29–3.12)
Anorectal symptoms								
No	1	1	1	1	1	1	1	1
Yes	17.16 (9.69–30.4)	17.25 (9.12–32.62)	9.09 (5.97–13.84)	8.95 (5.86–13.67)	12.02 (7.42–19.47)	14.8 (8.8–24.91)	7.93 (5.17–12.18)	9.53 (6.01–15.12)
HIV status								
Negative	1	1	1	1	1	1	1	1
Living with HIV	9.32 (5.88–14.76)	9.73 (5.51–17.2)	2.01 (1.34–3.02)	1.91 (1.21–2.99)	2.23 (1.44–3.44)	3.29 (1.96–5.53)	2.88 (1.95–4.26)	3.3 (2.13–5.12)

OR: odds ratio.

^a^ Adjusted for all the covariates shown in the table.

### Lymphogranuloma venereum genotyping

**Figure 2:**
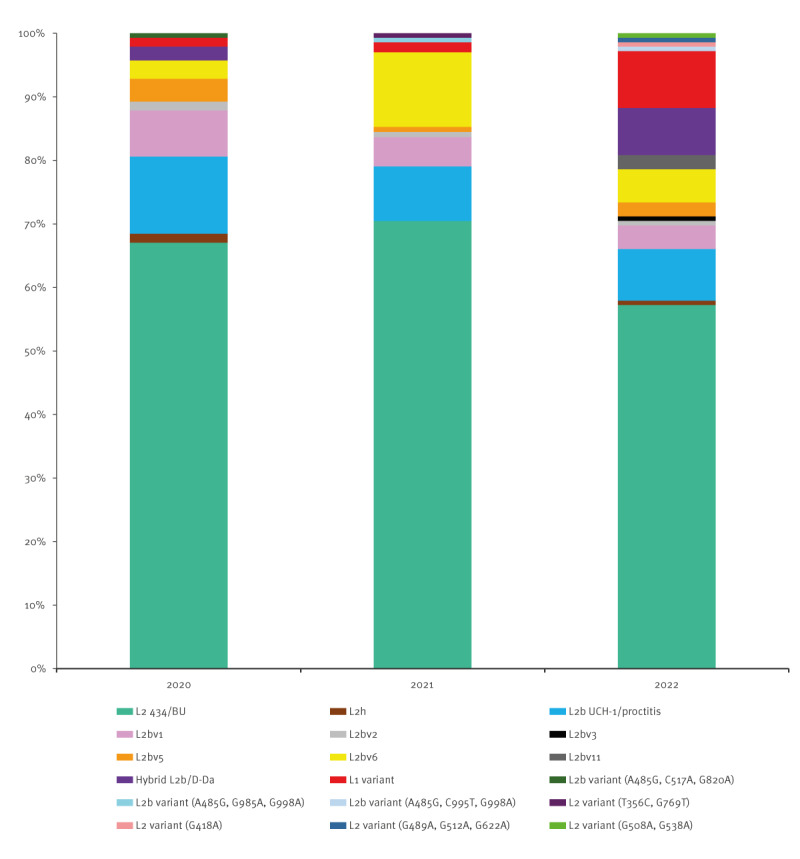
Distribution of lymphogranuloma venereum *omp*A genotypes in specimens, by year, France, 2020–2022 (n = 400)

Sequencing of *omp*A gene was successful for 94% (409/435) of LGV specimens. An L *omp*A genotype was identified in 400 of the 409 cases. We identified eighteen LGV *omp*A genotypes (Figure 2); for a list see Supplementary Table S5. A total of 64.5% (258/400) were of *omp*A genotype L2/434/Bu and 9.8% (39/400) were of *omp*A genotype L2b. We also identified sequences that varied from these by up to 3 bp: five *omp*A genotype L2 variants in seven specimens and nine *omp*A genotype L2b variants in 67 specimens. Among the latter, L2bv6 and L2bv1 were the most prevalent. We analysed the distribution of LGV *omp*A genotypes over time in France (Figure 2); the breakdown by year is appended in Supplementary Table S6. The percentage of *omp*A genotype L2b samples decreased from 12.4% (17/137) in 2020 to 8.1% (11/135) in 2022. An increase in L2bv6 was observed in 2021 (11.7%, 15/128), while L2bv1 decreased over time. The L2b/D-Da hybrid variant was identified in three specimens (2.2%, 3/137) in 2020 and then in 10 specimens in 2022 (7.4%, 10/135); it was not detected in 2021. The proportion of specimens with *omp*A genotype L1 variant increased markedly from 1.4% (2/137) in 2020 to 8.9% (12/135) in 2022.

For the new *omp*A genotype L2 and L2b variants described in this study, the substitutions compared with reference nucleotide sequences are indicated in parenthesis.

**Figure 3:**
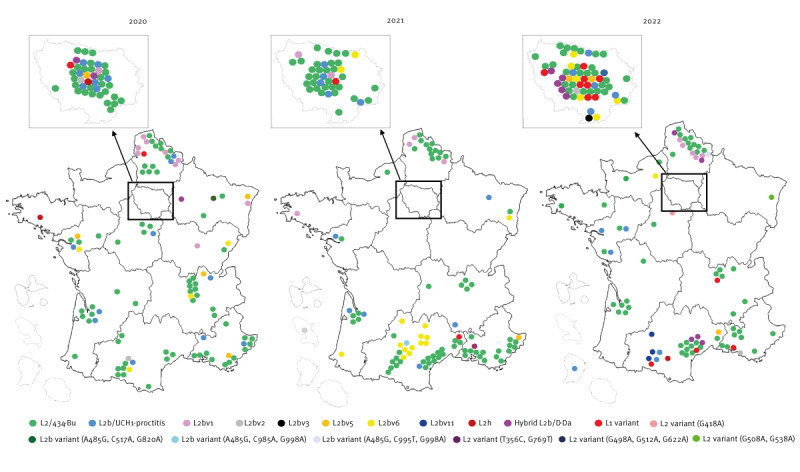
Distribution of lymphogranuloma venereum cases, metropolitan and overseas France, 2020–2022 (n = 400)

The spatial distribution of cases according to department of residence revealed that *omp*A genotypes L2/434/Bu and L2b were present throughout France during the 3-year survey period (Figure 3). By contrast, L2bv6 was identified mainly in one region of southern France in 2021, and only in the Paris region in 2022. The L2bv1 variant was mainly detected in the northern regions of France. In 2022, cases with the L1 variant did not cluster geographically but were distributed between Paris and southern France. The 10 cases with the L2b/D-Da hybrid variant were detected in three metropolitan regions of France. Of note, LGV *omp*A genotype diversity in the Paris region increased during the 3-year survey period.

The different circles correspond to LGV *omp*A genotypes identified in the study. Each colour represents a different LGV *omp*A genotype.

**Figure 4:**
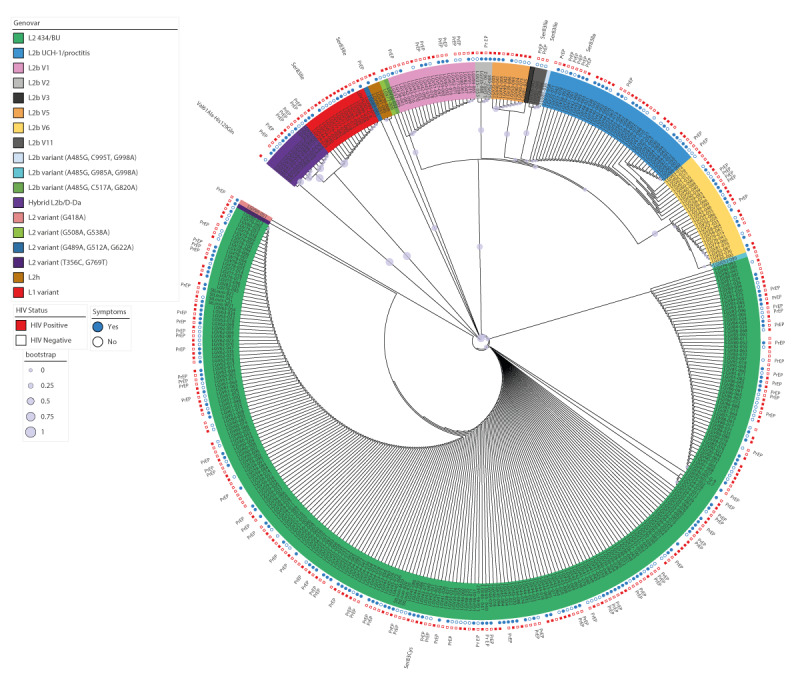
Maximum likelihood tree based on the *omp*A nucleotide sequence of the 400 lymphogranuloma venereum specimens collected during the 3-year national survey, France, 2020–2022 (n = 400)

We found no association between LGV *omp*A genotypes and anorectal symptoms, HIV status or PrEP use (Figure 4). Further details on case characteristics by *omp*A genotype are appended in Table S7. 

The evolutionary history was inferred by using the maximum likelihood method based on the Tamura 3-parameter model (T92 G + I model) [15] conducted in MEGA7 [16]. Branch support values were generated from 1,000 bootstrap replicates. The sequence of the *Chlamydia trachomatis* L2/434/Bu (AM8841176) was used as a reference. The phylogenetic tree is annotated with (from the centre to the periphery) the specimen name and, if available, the presence of anorectal symptoms, the HIV status, the presence of the GyrA mutation, and the PrEP use. Each LGV *omp*A genotype is represented by a different colour. The phylogenetic tree was displayed and annotated using iTOL (version 6.0).

In nine cases, *pmp*H gene amplification and *omp*A sequencing yielded discordant genotypes identifications, suggesting genetic exchange between L and non-L genovars. To confirm this hypothesis, whole genome sequencing would be needed which was not available for this study.

Of the 400 specimens with LGV *omp*A genotypes, the *gyr*A gene was successfully sequenced in 283 (70.8%) (Figure 4). Amino acid changes in the GyrA protein were identified in only seven samples. We detected the Ser83Cys change (*Escherichia coli* numbering) in one *omp*A genotype L2/434/Bu sample. One L2b/D-Da hybrid variant sample had two amino acid changes, Val61Ala and His129Gln, which have not been associated with fluoroquinolone resistance. The Ser83Ile mutation was found in one *omp*A genotype L2b sample in 2020 and two L1 variant samples from individuals living with HIV in the Paris region in 2022. Two of the three L2bv11 had the Ser83Ile amino acid change in the GyrA QRDR; *gyr*A gene was not amplified in the third case. The three L2bv11 samples were found in March 2022 in the same region of France among HIV-negative people (including two PrEP users), suggesting the circulation of this mutated strain.

## Discussion

This nationwide study showed that LGV positivity was stable in France from 2020 to 2022, with an increased detection of asymptomatic rectal LGV. More than half of the LGV infections were found in HIV-negative individuals, although there was a decreasing epidemiological trend over the study period.

In universal testing performed in France until 2015, LGV infection was detected mainly in MSM living with HIV [6]. In the 2020–2022 survey, most cases of LGV occurred in HIV-negative individuals, representing a major shift in the epidemiology of LGV in France. Such findings have been reported in EU/EEA countries that have implemented universal LGV testing of MSM irrespective of HIV status and anorectal symptoms. In the UK, the proportion of LGV among HIV-negative individuals was 58.4% in 2019; in Belgium and the Netherlands, this proportion was 29% and 49.3%, respectively, in 2017 [3,5,18]. In France, this increase was probably a result of the widespread use of PrEP (available since 2016) among HIV-negative individuals engaged in high-risk sexual activities. As PrEP administration requires regular STI testing, it provides more opportunities for LGV detection. Thus, the positivity for LGV among French PrEP users was 18.7% (91/486) in 2017 and did not increase during the 3-year period of the present survey [19]. In Belgium, LGV cases among HIV-negative men began to be detected before the introduction of PrEP and did not increase thereafter [18]. We also observed a higher proportion of LGV cases among people living with HIV between 2020 and 2022, but this increase was not significant (p = 0.5). This could be linked to an increase in HIV testing in 2021 and 2022, after a sharp drop in 2020 because of the COVID-19 pandemic. In 2021 and 2022, the highest number of HIV-positive tests was reported in the Paris region, which could explain the increased number of LGV cases in this region.

French data from 2010 to 2015 revealed that 94% of LGV-positive individuals reported anorectal symptoms [6]. Based on the same testing methodology, we report for the first time an increase in asymptomatic LGV cases in France, in HIV-negative individuals as well as in people living with HIV. A similar observation was reported for the Netherlands [5]. Our findings showed an even higher percentage of asymptomatic LGV cases than three studies conducted in other EU/EEA countries [4,5,20]. However, LGV diagnosis in France continues to be associated with living with HIV, anorectal symptoms and residence in the Paris region.

In the present study, participants were younger in 2021 than in 2020 and 2022. As French public health data showed that between 2019 and 2021, the screening rate for *C. trachomatis* infection was higher among men younger than 26 years than among older men [21], we hypothesise that young people may have been more inclined to postpone visiting any medical centre and delayed seeking healthcare during the Covid-19 pandemic, which would allow untreated infections to spread. In 2021, these individuals may have returned to the STI screening centres.

Genotyping showed that the *omp*A genotype L2/434/Bu was predominant in France, confirming a previous report and in line with other findings from EU/EEA countries [7,8,22,23]. The *omp*A genotype L2b decreased continuously over the 3-year survey period. The diversity of LGV *omp*A genotypes was greater than reported previously [8]. The *omp*A genotype L2b variants accounted for 16% of LGV cases; L2bv1 and L2bv6 predominated. The latter have been reported sporadically in Austria, the Netherlands, Spain, Sweden and the UK [23-25]. The spatiotemporal distribution of the LGV *omp*A genotypes identified in this study provides insight into the endemic situation and transmission networks in France. For instance, the diversity of *omp*A genotypes increased in the Paris region, indicating the presence of a very active transmission chain. Moreover, the sudden increase in L2bv6 cases in a region in the south of France suggests that new outbreaks occurred in association with sexual networks. The L2bv6 variant was no longer detected in this region in 2022 and was instead found mainly in the Paris region. Cases with L2bv1 decreased over time but remained localised in northern France. The L2b/D-Da hybrid variant emerged throughout France in 2022. This strain was detected during a proctitis-associated LGV outbreak mainly in HIV-positive MSM in 2017 in Portugal [26]. The outbreak-causing L2b/D-Da hybrid *C. trachomatis* strain resulted from the genetic transfer of a ca 4.2 kbp genomic fragment (transferring 240 SNPs, 76 of which were within *omp*A) from a D/Da strain to a typical proctitis-associated L2b strain. All the recombinants were identical [26]. This variant accounted for 5% of LGV cases in the UK from 2018 to 2019 and 31.5% of LGV cases in Italy from 2019 to 2022 [7,9]. In Portugal, its prevalence decreased from 23.1% in 2017 to 3.1% in 2019 [22]. In France, the proportion of this hybrid variant was low in 2017 (4.6%, 4/87) and 2018 (4.1%, 6/146) and increased in 2022 (7.4%, 10/125) [27]. In our study, the L2b/D-Da hybrid variant was found in HIV-negative people and also in those without anorectal symptoms. We did not have the information necessary to establish an epidemiological link between these cases. The proportion of the *omp*A genotype L1 variant was unexpectedly large (8.9%) in 2022; this occurrence was not associated with clinical factors (HIV status or presence of anorectal symptoms) or the spatial distribution. A small L1 cluster was described in Seattle in the 1980s but did not spread further until its recent description in Argentina, where it accounted for 20.5% of symptomatic LGV cases [28,29]. No increase in the *omp*A genotype L1 variant has yet been reported in Europe [7,9,22,23,25].

Using whole genome sequencing, Borges et al. reported L2b/D-Da strains with a Ser83Ile amino acid change in the DNA gyrase subunit A [10]. In vitro, this substitution was associated with fluoroquinolone resistance in the *C. trachomatis* L2 reference strain [11]. In the present study, this mutation was identified in two *omp*A genotype L1 variant samples from the Paris region and in three L2bv11 samples from a southern French region in 2022, suggesting the circulation of fluoroquinolone-resistant strains in these regions. The clinical implications of fluoroquinolone-resistant strains are unknown because these antibiotics are not first-line drugs for *C. trachomatis* treatment.

This study has several limitations. Firstly, as laboratories send the specimens voluntarily, the results are not based on all LGV cases occurring in France. Nevertheless, 59 laboratories participated each year, yielding comparable data over the 3-year period. In some regions, the surveillance network might be improved. Secondly, the survey duration was 3 months per year. Although the study took place in autumn 2020 and spring 2021 and 2022, there was no statistically significant difference in LGV positivity by month. Similar numbers of samples were received in 2020 and 2021, and increased in 2022, showing the interest of laboratories to participate in this study. Thirdly, the rate of missing data was 6%, 8% and 12% for HIV status and 16%, 26% and 17% for anorectal symptoms in 2020, 2021 and 2022, respectively. Fourthly, we used an in-house real-time PCR for LGV diagnosis targeting the *pmp*H gene which could have lower sensitivity than those of the recent commercial assays [12]. However, we recently showed that the four commercial assays currently available for LGV detection have similar performances than in our in-house real-time PCR for LGV diagnosis in anorectal *C. trachomatis*-positive specimens [30]. Finally, the 2020 and 2021 surveys were carried out during the COVID-19 pandemic, when different infection control measures, including social distancing, were in place. Nonetheless, one study in Spain showed that the restrictions, including lockdown, have had no impact on reducing LGV transmission [31].

## Conclusion

In France, selective LGV testing of people living with HIV and/or with anorectal symptoms, implemented following the analysis of LGV data from 2010 to 2015, should no longer be performed. Among LGV cases, the increases in asymptomatic cases and in the proportion of HIV-negative individuals indicate the need for universal LGV testing. Our findings also provide insight into the diversity and spatiotemporal distribution of circulating LGV *omp*A genotypes in France. This improves our understanding of the evolution and dissemination of LGV strains linked to sexual networks. 

## Supplementary Data

306000 Supplement
