# Supplementary material for: Anorectal lymphogranuloma venereum among men who have sex with men: a 3-year nationwide survey, France, 2020 to 2022
Source: Euro Surveill. 2024 May 9;29(19):2300520. doi: 10.2807/1560-7917.ES.2024.29.19.2300520 (PMC11083974; doi:10.2807/1560-7917.ES.2024.29.19.2300520)
Supplement: Supplement [file 23-00520_PEUCHANT_Supplement.pdf]

Supplementary material

This supplementary material is hosted by *Eurosurveillance* as supporting information alongside the article "*Anorectal lymphogranuloma venereum among men who have sex with men: a 3-year nationwide survey, France, 2020 to 2022*", on behalf of the authors, who remain responsible for the accuracy and appropriateness of the content. The same standards for ethics, copyright, attributions and permissions as for the article apply. Supplements are not edited by *Eurosurveillance* and the journal is not responsible for the maintenance of any links or email addresses provided therein.

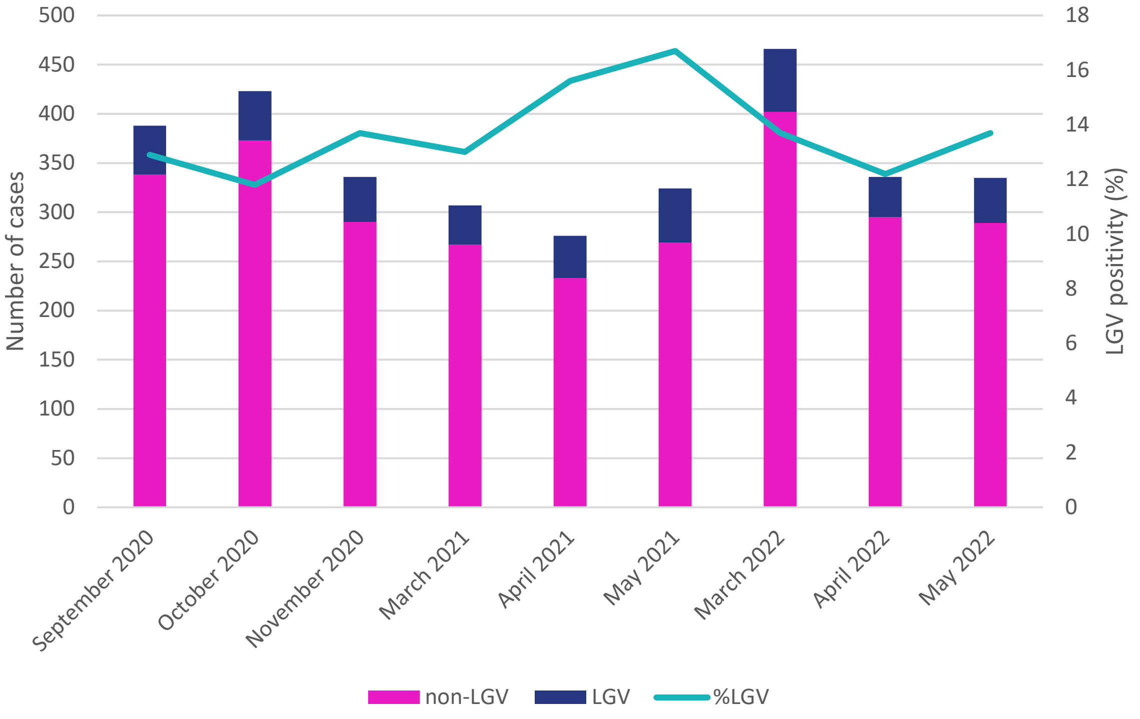

Figure S1. Number of LGV and non-LGV cases, and LGV positivity each month of the study.

Table S1. Tabular overview of changes in the screening policies of *Chlamydia trachomatis* infection over time in some European countries.

Country	Screening policies over time
**The Netherlands** [1]	**National STI surveillance system of Centres for Sexual Health (CSH)**<br><br>**Until 2015**: **selective testing**: rectal *Chlamydia trachomatis* testing for only MSM who reported having had receptive anal sex; among those with a positive rectal *C. trachomatis* test result, LGV testing was recommended, in particular for HIV-positive MSM or MSM presenting with LGV-related symptoms<br><br>**Since 2015**: **Universal** rectal chlamydia testing for all MSM and universal LGV testing in all rectal *C. trachomatis*-positive MSM
**United Kingdom**	**2010 - *Chlamydia trachomatis* UK testing guidelines** [2]<br>• All MSM positive for rectal *Chlamydia trachomatis* with any rectal symptoms<br>• Contacts of confirmed LGV cases<br>**2013 - UK National Guideline for the management of LGV** [3]<br>• All individuals with *Chlamydia trachomatis* positive rectal and pharyngeal sites who exhibit symptoms consistent with LGV<br>• Contacts of confirmed LGV cases<br>**2015 - UK national guideline for the management of infection with *Chlamydia trachomatis*** [4]<br>• All individuals with symptoms consistent with LGV<br>• All MSM living with HIV with positive *Chlamydia trachomatis* at any site regardless of LGV symptoms.
**Belgium**	**2011 :** All MSM positive for rectal *Chlamydia trachomatis* with any rectal symptoms<br>**2018 :** Testing all positive anorectal *Chlamydia trachomatis* samples of MSM for LGV confirmation irrespective of their HIV status [5]

1.Van Aar F, Kroone MM, de Vries HJ, Götz HM, van Benthem BH. Increasing trends of lymphogranuloma venereum among HIV-negative and asymptomatic men who have sex with men, the Netherlands, 2011 to 2017. Eurosurveillance. 2020;25(14):1900377.
2. BASHH (2010). *Chlamydia trachomatis* UK testing guidelines.
3. White J, O'Farrell N, Daniels D. 2013 UK National Guideline for the management of Lymphogranuloma venereum. Clinical Effectiveness Group of the British Association for Sexual Health and HIV (CEG/BASHH) Guideline development group. Int J STD AIDS. 2013;24(8):593-601
4. Nwokolo NC, Dragovic B, Patel S, Tong CYW, Barker G, Radcliffe K. 2015 UK national guideline for the management of infection with *Chlamydia trachomatis*. Int J STD AIDS 2016. 27(4): p 251-67.
5. Jespers V, Stordeur S, Desomer A, et al. Sexually Transmitted Infections in Primary Care Consultations: Development of an Online Tool to Guide Healthcare Practitioners. Brussels. 2019. Available at: https://www.sti. kce.be/en/. Accessed June 15, 2020.

Table S2. Number of specimens and LGV/non-LGV cases in Paris region, other metropolitan French regions with detail by region, and overseas territories.

	2020			2021			2022		
	N (total)	LGV n (%)	non-LGV n (%)	N (total)	LGV n (%)	non-LGV n (%)	N (total)	LGV n (%)	non-LGV n (%)
**Overseas territories**	**24**	**0**	**24 (100)**	**12**	**1 (8.3)**	**11 (91.7)**	**41**	**1 (2.4)**	**40 (97.6)**
**Paris region**	**383**	**47 (12.3)**	**336 (87,7)**	**246**	**42 (17.1)**	**204 (82.9)**	**331**	**61 (18.4)**	**270 (81.6)**
**Other metropolitan French regions**	**740**	**99 (13.4)**	**641 (86.6)**	**649**	**95 (14.6)**	**554 (85.4)**	**765**	**89 (11.6)**	**676 (88.4)**
Detail by region									
Hauts-de-France	**124**	24 (24)	100 (76)	**84**	15 (17.9)	69 (82.1)	**145**	21 (14.5)	124 (85.5)
Normandie	**24**	0	24 (100)	**27**	1 (3.7)	26 (96.3)	**33**	3 (9.1)	30 (90.9)
Bretagne	**19**	0	19 (100)	**25**	1 (4)	24 (96)	**30**	2 (6.7)	28 (93.3)
Grand-Est	**46**	5 (18.9)	41 (8.9)	**42**	3 (7.1)	39 (92.8)	**20**	1 (5)	19 (95)
Bourgogne-Franche-Comté	**19**	4 (21.1)	15 (78.9)	**18**	0	18 (100)	**23**	1 (4.3)	22 (9.6)
Centre-Val-de-Loire	**17**	4 (23.5)	13 (76.5)	**19**	2 (10.5)	17 (89.5)	**25**	3 (12)	22 (88)
Pays de la Loire	**51**	7 (13.7)	44 (86.3)	**32**	2 (6.3)	30 (93.7)	**77**	7 (9.1)	70 (90.9)
Nouvelle-Aquitaine	**50**	10 (20)	40 (80)	**33**	7 (21.2)	26 (78.8)	**57**	7 (12.3)	50 (87.7)
Occitanie	**150**	18 (12)	132 (88)	**168**	36 (21.4)	132 (78.6)	**166**	25 (15.1)	141 (84.9)
Auvergne-Rhône-Alpes	**124**	13 (10.5)	111 (89.5)	**107**	7 (6.5)	100 (93.5)	**132**	8 (6.1)	124 (93.9)
Provence-Alpes-Côtes-D'azur	**114**	14 (12.3)	100 (87.7)	**93**	21 (22.6)	72 (77.4)	**56**	11 (19.6)	45 (80.4)

Table S3. Anorectal symptoms in LGV cases according to HIV status and by year.

	**2020** n (%)	**2021** n (%)	**2022** n (%)	p-value
**Individuals living with HIV[a]**	N=45	N=46	N=64	
Symptomatic	29 (64.4)	19 (41.3)	25 (39.1)	**0.02**
Asymptomatic	16 (35.6)	27 (58.7)	39 (60.9)	
**HIV-negative[b] individuals**	**N=75**	**N=52**	**N=56**	
Symptomatic	47 (62.7)	37 (71.2)	31 (55.4)	0.24
Asymptomatic	28 (37.3)	15 (28.8)	25 (44.6)	

[a] Data were not available for 11 patients in 2020 and 7 patients in 2021.
[b] Data were not available for 6 patients in 2020, 22 patients in 2021 and 13 patients in 2022.

Table S4. Anorectal symptoms among HIV-negative individuals with LGV according to PrEP use and by year.

	**2020** n (%)	**2021** n (%)	**2022** n (%)	p-value
**PrEP users**	N=49	N=38	N=36	
Symptomatic	30 (61.2)	28 (73.7)	18 (50)	0.11
Asymptomatic	19 (38.8)	10 (26.3)	18 (50)	
**HIV-negative individuals not on PrEP[a]**	N=19	N=11	N=13	
Symptomatic	14 (73.7)	7 (63.6)	6 (46.2)	0.27
Asymptomatic	5 (26.3)	4 (36.7)	7 (53.8)	

[a] Data were not available for one patient in 2020 and one patient in 2022.

Table S5. *omp*A-genotypes relevant to the study

Nomenclature of *omp*A-genotype	Nucleotide change from reference L2/434/Bu (AM884176)	Amino Acide changes from reference L2/434/Bu (AM884176)
L2/434-Bu	-	-
L2b UCH-1/proctitis	A485G	N162S
L2bV1	A485G, C517A	N162S, L173I
L2bV2	A485G, A515C	N162S, K172T
L2bV3	A485G, C493A	N162S, H165N
L2bV5	G271A, A485G, C493A	A91T, N162S, H165N
L2bV6	A485G, G998A	N162S, S333N
L2bV11	A485G, G1000A	N162S, A334T
L2b variant	A485G, C995T, G998A	N162S, D329N, S333N
L2b variant	A485G, G985A, G998A	N162S, A332V, S333N
L2b variant	A485G, C517A, G820A	N162S, L173I, A274T
L2h	A997G	S333G
L2 variant	G418A	G140R
L2 variant	G508A, G538A	D170N, D180N
L2 variant	G489A, G512A, G622A	S171N, A208T
L2 variant	T356C, G769T	F119F, D257Y
Hybrid L2b/D-Da	-	-
L1 variant	-	-

Table S6. LGV *omp*A-genotypes identified according to the year of the study.

Number of LGV *omp*A-genotypes cases	2020<br>N=137	2021<br>N=128	2022<br>N=135
	N (%)	N (%)	N (%)
L2/434/Bu	91 (66.4)	90 (70.31)	77 (57)
L2 variant (T356C, G769T)*	0	1 (0.8)	0
L2 variant (G418A)*	0	0	1 (0.7)
L2 variant (G498A, G512A, G622A)*	0	0	1 (0.7)
L2 variant (G508A, G538A)*	0	0	1 (0.7)
L2h	2 (1.4)	0	1 (0.7)
L2b/UCH-1 proctitis	17 (12.2)	11 (8.6)	11 (8.1)
L2bv1	10 (7.3)	6 (4.7)	5 (3.7)
L2bv2	2 (1.4)	1 (0.8)	1 (0.7)
L2bv3	0	0	1 (0.7)
L2bv5	5 (3.6)	1 (0.8)	3 (2.2)
L2bv6	4 (3.9)	15 (11.7)	7 (5.2)
L2bv11	0	0	3 (2.2)
L2b variant (A485G, C517A; G820A)*	1 (0.8)	0	0
L2b variant (A485G G985A, G998A)*	0	1 (0.8)	0
L2b variant (A485G C995T, G998A)*	0	0	1 (0.7)
L2b/D-Da hybrid variant	3 (2.2)	0	10 (7.4)
L1 variant	2 (1.4)	2 (1.6)	12 (8.9)

*new *omp*A-genotype L2 and L2b variants described in this study; substitutions compared to reference nucleotide sequences are indicated in parenthesis.

Genbank accession number: L2/434/Bu: AM884176; L2a/UW-396: AB915594, L2b/UCH-1 proctitis: AM884177; L2h: MH253042; L2bv1: JX971936; L2bv2: KU518893; L2bv3: KU518894; L2bv4: KU518892; L2bv5: MH253040; L2bv6: MH253041; L2bv11: LR882858; L1 variant: MN563611; L2b/D-Da hybrid variant: MN094864.

Table S7. Clinical and epidemiological characteristics of LGV-positive individuals stratified by LGV *omp*A-genotype, 2020-2022. Only LGV *omp*A-genotypes identified more than once in the study are presented in this table.

	L2/434/Bu (N=258)	L2h (N=3)	L2b/UCH-1 proctitis (N=39)	L2bv1 (N=21)	L2bv2 (N=4)	L2bv5 (N=9)	L2bv6 (N=26)	L2bv11 (N=3)	L2b/D-Da hybrid (N=13)	L1 variant (N=16)
	n (%)	n (%)	n (%)	n (%)	n (%)	n (%)	n (%)	n (%)	n (%)	n (%)
**Anorectal symptoms**	N=205	N=2	N=32	N=15	N=4	N=8	N=21	N=3	N=12	N=14
Yes	121 (59)	2 (100)	19 (59.4)	8 (53.3)	3 (75)	4 (50)	13 (61.9)	0	6 (50)	9 (64.3)
No	84 (41)	0	13 (40.6)	7 (46.7)	1 (25)	4 (50)	8 (38.1)	3 (100)	6 (50)	5 (35.7)
**HIV status**	N=236	N=3	N=35	N=20	N=4	N=9	N=23	N=3	N=11	N=15
Positive	99 (41.9)	2 (66.7)	16 (45.7)	5 (25)	2 (50)	7 (77.8)	12 (52.2)	0	5 (45.5)	10 (66.7)
PrEP users	76 (32.2)	1 (33.3)	9 (25.7)	7 (35)	1 (25)	0	5 (21.7)	2 (66.7)	5 (45.5)	2 (13.3)
Negative (not using PrEP or PrEP use unknown)	61 (25.8)	0	10 (28.6)	8 (40)	1 (25)	2 (22.2)	6 (26.1)	1 (33.3)	1 (0.1)	3 (20)
**Residence**	N=258	N=3	N=39	N=21	N=4	N=9	N=26	N=3	N=13	N=16
Paris region	84 (32.6)	1 (33.3)	17 (43.6)	3 (14.3)	1 (33.3)	3 (33.3)	8 (44.4)	0	7 (53.8)	10 (62.5)
Regions other than Paris	174 (67.4)	2 (66.7)	21 (53.8)	18 (85.7)	2 (66.7)	6 (66.7)	18 (69.2)	3 (100)	6 (46.2)	6 (37.5)
Overseas territories	0	0	1 (2.6)	0	1	0	0	0	0	0
**GyrA Protein**	N=180	N=2	N=31	N=17	N=2	N=6	N=17	N=2	N=8	N=14
No amino acid change	179 (99.4)	2 (100)	30 (96.8)	17 (100)	2 (100)	6 (100)	17 (100)	0	7 (87.5)	12 (85.7)
Amino acid change[a]	Ser83Cys n=1 (0.6)	0	Ser83Ile n=1 (3.2)	0	0	0	0	Ser83Ile n=2 (100)	Val61Ala-His129Gln n=1 (12.5)	Ser83Ile n=2 (14.3)

[a]*Escherichia coli* numbering.
